# Unveiling of brain transcriptome of masked palm civet (*Paguma larvata*) with chronic infection of *Toxoplasma gondii*

**DOI:** 10.1186/s13071-022-05378-5

**Published:** 2022-07-24

**Authors:** Hao Yuan, Xiu-Xiang Zhang, Zi-Peng Yang, Xiao-Hu Wang, Yasser S. Mahmmod, Pian Zhang, Zi-Jing Yan, Yan-Yun Wang, Zhao-Wen Ren, Qing-Yong Guo, Zi-Guo Yuan

**Affiliations:** 1 College of Veterinary Medicine, Xinjiang Agricultural University, Urumqi, 830052 Xinjiang People’s Republic of China; 2 College of Veterinary Medicine, South China Agricultural University, Guangzhou, 510642 Guangdong People’s Republic of China; 3 Key Laboratory of Zoonosis Prevention and Control of Guangdong Province, Guangzhou, 510642 People’s Republic of China; 4 Key Laboratory of Zoonosis of Ministry of Agriculture and Rural Affairs, South China Agricultural University, Guangzhou, 510642 Guangdong People’s Republic of China; 5 Institute of Animal Health, Guangdong Academy of Agricultural Sciences, Guangzhou, 510640 Guangdong People’s Republic of China; 6 Infectious Diseases, Department of Animal Medicine, Faculty of Veterinary Medicine, Zagazig University, Zagazig, 44511 Sharika Egypt; 7 Veterinary Sciences Division, Faculty of Health Sciences, Higher Colleges of Technology, 17155- Al Ain, Abu Dhabi, United Arab Emirates; 8 College of Agriculture, South China Agricultural University, Guangzhou, 510642 Guangdong People’s Republic of China

**Keywords:** *Toxoplasma gondii*, Masked palm civet, Gene expression, RNA-Seq

## Abstract

**Background:**

The aim of this study was to gain an understanding of the transcriptomic changes that occur in a wild species when infected with *Toxoplasma gondii*. The masked palm civet, an artifically domesticated animal, was used as the model of a wild species. Transcriptome analysis was used to study alterations in gene expression in the domesticated masked palm civet after chronic infection with *T. gondii*.

**Methods:**

Masked palm civets were infected with 10^5^ T*. gondii* cysts and their brain tissue collected after 4 months of infection. RNA sequencing (RNA-Seq) was used to gain insight into the spectrum of genes that were differentially expressed due to infection. Quantitative reverse-transcription PCR (qRT-PCR) was also used to validate the level of expression of a set of differentially expressed genes (DEGs) obtained by sequencing.

**Results:**

DEGs were screened from the sequencing results and analyzed. A total of 2808 DEGs were detected, of which 860 were upregulated and 1948 were downregulated. RNA-Seq results were confirmed by qRT-PCR. DEGs were mainly enriched in cellular process and metabolic process based on gene ontology enrichment analysis. Kyoto Encyclopedia of Genes and Genomes pathway analysis showed that transcriptional changes in the brain of infected masked palm civets evolved over the course of infection and that DEGs were mainly enriched in the signal transduction, immune system processes, transport and catabolic pathways. Finally, 10 essential driving genes were identified from the immune signaling pathway.

**Conclusions:**

This study revealed novel host genes which may provide target genes for the development of new therapeutics and detection methods for *T. gondii* infection in wild animals.

**Graphical Abstract:**

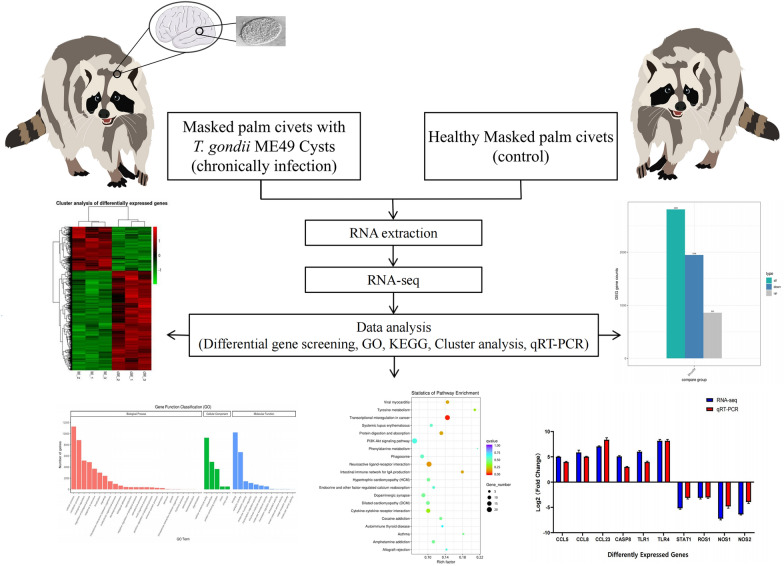

**Supplementary Information:**

The online version contains supplementary material available at 10.1186/s13071-022-05378-5.

## Background

*Toxoplasma gondii* is a protozoan parasite that infects exclusively eukaryotic cells. Toxoplasmosis is a major parasitic zoonosis caused by *T. gondii* [1]. The accidental consumption of food or water containing *T. gondii* oocysts is one of the routes by which organisms become infected with *T. gondii*. Toxoplasmosis can cause severe clinical symptoms and even death in infants, pregnant women and immunocompromised individuals [2, 3]. This parasite can infect all host tissues, but the central nervous system and muscles are the predilection sites [4]. Tachyzoites of *T. gondii* can invade almost all nucleated cells but have a particular affinity for nerve cells, resulting in *T. gondii* encephalitis [5]. Epidemiological surveys have shown that about 30% of the world's population is potentially infected with *T. gondii* [6]. Among the causes of death due to *T. gondii*, 90% are due to encephalitis, with a high proportion of secondary paralysis [7]. In addition, *T. gondii* is also one of the main factors affecting the health of patients with human immunodeficiency virus (HIV) [8]. Studies have shown that 65% of patients with HIV die from the re-activation of *T. gondii* infection in the first year after diagnosis [9]. Chronic *T. gondii* infection can also cause neurological diseases, such as schizophrenia and depression [10]. Felids can be both definitive and intermediate hosts of *T. gondii*. The ME49 strain (type II) of either moderate or low virulence to animal hosts is able to control the acute phase of the disease and able to establish chronic infections, and is the type most frequently associated with human and animal disease [11].

The masked palm civet, *Paguma larvata* (Mammalia: Carnivora: Viverridae), is a carnivore species widely distributed in the vast area south of the Yellow River in China [12]. Once served as food on plates in China, masked palm civets have become another transmission route of many zoonotic parasites, bacteria and viruses, such as *T. gondii*, *Enterocytozoon bieneusi*, *Bartonella henselae*, *Salmonella enterica*, *Campylobacter* spp. and *Cryptosporidium* spp. [13–16]. Studies on the changes in gene expression in wild animals following pathogen infection will provide critical information for analyzing host resistance strategies.

In recent years, various “omics” methods have been developed and used in studies to explore the mechanism by which *T. gondii* invades the brain [17–19]. Based on the results of these studies, it can be concluded that pathogenesis of the host nervous system in *T. gondii* infection is driven by complex molecular processes and pathway networks [20–22]. The purpose of this study was to investigate the pathological changes in the brain of the masked palm civet chronically infected with *T. gondii*. The secondary purpose was to investigate the genes found to be associated with infection, and the patterns of these genes in the brain tissue of animals infected with *T. gondii* using transcriptome analysis. Transcriptome analysis of the mechanism by which *T. gondii* enters the brain can reveal different aspects of the host's immune response, prevention of infection and control mechanisms of wild species chronically infected with this protozoan.

## Methods

### Experimental animals

Six 3- to 4-month-old masked palm civets were purchased from the Jiahe Special Breeding Station in Shaoguan (Guangdong, China). All masked palm civets were confirmed to be negative for *T. gondii* using the modified agglutination test. For 3 weeks before the experiment, the animals were fed on a commercial diet provided with potable water according to the daily energy requirements of experimental animals.

### Sample collection

*Toxoplasma gondii* ME49 strain (genotype #2) was cultured in Kunming mice in the Parasitology Laboratory of South China Agricultural University (Guangdong, China). Brain cysts of *T. gondii* were examined microscopically and their number adjusted to 10^3^ cysts/ml in phosphate-buffered saline (PBS). Six masked civets were average divided into two groups, control group and intected group. Animals in the experimental group were infected by intragastric inoculation with 10^5^ cysts of *T. gondii* in 1 ml sterile PBS. Animals in the control group remained uninfected and received 1 ml of sterile PBS only. After 4 months of experimental infection, the aseptic brain tissue of the experimental and control animals collected. The collected tissue samples were washed in saltwater, rapidly frozen in liquid nitrogen and stored at − 80 °C until further treatment.

### Detection of *T. gondii*

DNA was extracted from the samples of brain tissue from the masked civets using the TIANamp Genomic DNA Kit (TianGen, Beijing, China). In an earlier study, Ye et al. [23] obtained the primer sequences against the *T. gondii* B1 gene and the bradyzoite antigen-1 (BAG1). The B1 gene primers (forward: 5′-TCTTTAAAGCGTTCGTGTC-3′; reverse: 5′-GGAACTGCATCCGTTCATGAG-3′) and BAG1 gene primers (forward: 5′-AGTCGACAACGGAGCCATCGTTA-3′; reverse: 5′-CCTTGATCGTGACACGTAGAACG-3′) were amplified for the quantitative reverse-transcription PCR (qRT-PCR) assays.

### RNA extraction and identification

TRIzol reagent was utilized to extract total RNA from brain tissue. Approximately 50 mg of the sample was ground to powder with an appropriate amount of liquid nitrogen, and then 1 ml of TRIzol reagent homogenate was added [24]. The purity of the RNA was determined using the Nanodrop 2000 spectrophotometer (Thermo Fisher Scientific, Waltham, MA, USA). Degradation and contamination of RNA was examined on a 2.5% agarose gel using a Qubit®3.0 fluorometer (Thermo Fisher Scientific). The integrity and total RNA amount were accurately detected on a Agilent 2100 bioanalyzer (Agilent Technologies Inc., Santa Clara, CA, USA).

### Library preparation for transcriptome sequencing

RNA sample preparations utilized RNA as input material. Briefly, messenger RNA (mRNA) was purified from total extracted RNA using poly-oligo-attached magnetic beads, and then the mRNA was fragmented using divalent cations under elevated temperature, in First Strand Synthesis Reaction Buffer (5×) (Thermo Fisher Scientific). The first-strand cDNA was synthesized using random hexamer primer and the M-MuLV Reverse Transcriptase enzyme, and then degraded by RNAse H treatment. The second-strand cDNA was synthesized using DNA Polymerase I and dNTP. The remaining overhangs were converted into blunt ends via treatment with exonuclease/polymerase after the addition of 3' ends of DNA fragments. The adaptors ligated the preparation of the hybridization step with hairpin loop structures. cDNA fragments with a preferred length of 370–420 bp were selected, and the library fragments were purified by the AMPure XP system (Beckman Coulter Inc., Brea, CA, USA). PCR assays were performed with Phusion High-Fidelity DNA polymerase, Universal PCR primers and Index (X) Primer. Finally, the PCR products were purified (AMPure XP System; Beckman Coulter Inc.), and the library quality was assessed on the Qubit2.0 fluorometer (Agilent Bioanalyzer 2100 System; Agilent Technologies Inc.) and by qRT-PCR.

### Data analysis

In-house Perl scripts were first used to obtain raw data (raw reads) in the FASTq format. In this step, clean data could be obtained (clean reads) by removing reads containing adapters, N base and low-quality reads from the raw data. Simultaneously, Q20, Q30, GC content and clear data were calculated. All downstream analyses were based on clean data of high quality [25].

#### Hierarchical clustering

The co-expression patterns of genes at different time points after infection were determined by cluster analysis of differentially expressed genes (DEGs). Genes with similar expression patterns were grouped into clusters. K-means clustering was achieved based on the relative expression level of DEGs log2 (fold change [FC]) [26].

Samples used for RNA sequencing were validated, and Pearson correlation analysis was used to assess the correlation of gene expression levels between samples. The square of Pearson correlation coefficient (*R*^2^) < 0.92 was defined as the optimal sampling selection and experimental condition [27].

The DESeq2R software package (version 1.20.0) was used to analyze differential expression between the two groups (experimental and control). The Benjamini–Hochberg method was used to adjust the* P*-value to regulate the error detection rate. The genes with adjusted* P* < 0.05 found by the DESeq2 software were designated as different genes [28, 29].

#### Gene ontology enrichment and Kyoto Encyclopedia of Genes and Genomes analysis

We used GOseq and KOBAS software for gene ontology (GO) functional enrichment analysis, Kyoto Encyclopedia of Genes and Genomes (KEGG) annotation and pathway enrichment analysis of differential gene sets. Regulation of false discovery rate < 0.05 and *P* < 0.05 were considered to be a significant enrichment pathway.

#### qRT-PCR validation of data

A sample of total RNA of approximately 1 ug was taken for further study based on the measured concentration. A capacity RNA kit was then used to synthesize cDNA by reverse transcription (Vazyme, Nanjing, China). qRT-PCR gene primers were designed using Premier 5.0 software. The components were mixed in advance using SYBR qPCR Master Mix (Vazymea), and the samples were then placed in a Roche LifeCycler® 96 real-time system for PCR cycling, with the cycling regimen consisting of 1 cycle at 95 °C for 30 s, followed by 40 cycles at 95 °C for 10 s and 60 °C for 30 s. Three independent replicates were used for each sample. β-Actin was used as an internal standard for gene expression normalization. The blank control group was taken as the reference sample and set as 1. Additional relative quantification of the target mRNA was performed using the cycle threshold (Ct) 2^−∆∆Ct^ method. The genes and primers used in qRT-PCR are shown in Table 1.

**Table 1:** Gene names and primers used in the quantitative reverse-transcription PCR analysis

Gene^a^	Sequence (5′-3′)	Primer length (mer)
*CCL5*	F: CCAGCAGTCGTCTTTGTCAC	20
	R: CTCTGGGTTGGCACACACTT	20
*CCL8*	F: TGGAGAGCTACACAAGAATCACC	23
	R: TGGTCCAGATGCTTCATGGAA	21
*CCL23*	F: CATCTCCTACACCCCACGAAG	22
	R: GGGTTGGCACAGAAACGTC	21
*CASP8*	F: TTTCTGCCTACAGGGTCATGC	21
	R: TGTCCAACTTTCCTTCTCCCA	21
*TLR1*	F: TTCAAACGTGAAGCTACAGGG	21
	R: CCGAACACATCGCTGACAACT	21
*TLR4*	F: AGACCTGTCCCTGAACCCTAT	21
	R: CGATGGACTTCTAAACCAGCCA	22
*STAT1*	F: CAGCTTGACTCAAAATTCCTGGA	23
	R: TGAAGATTACGCTTGCTTTTCCT	23
*ROS1*	F: GGCTGCCTATGGATTTCTGTG	21
	R: GCTGCTGGCCCAGATTAGTT	20
*NOS1*	F: TTCCCTCTCGCCAAAGAGTTT	21
	R: AAGTGCTAGTGGTGTCGATCT	21
*NOS2*	F: TTCAGTATCACAACCTCAGCAAG	23
	R: TGGACCTGCAAGTTAAAATCCC	22

*F* Forward, R reverse

^a^See Table 2 for a full description

## Results

### Confirmation of *T. gondii* infection

A 194-bp fragment of the B1 gene and a 200-bp fragment of the BAG1 gene were successfully amplified by PCR and qRT-PCR, respectively. The presence of these fragments was additional confirmation of the existence of *T. gondii* cysts in brain tissue.

###  RNA-sequencing data

The RNA templates of the RNA libraries in both the infection group and control group were all > 8.0. The high-quality of the RNA libraries facilitated the analysis of subsequent data.

According to the quality evaluation results obtained, 130,016,821 clean reads remained after removing joints and low-quality reads. A total of 2808 DEGs were detected and screened in the brain tissue taken from the masked palm civets, of these 860 were upregulated DEGs and 1948 were downregulated DEGs. Compared with the reference genome, this could reach 82.5%. The GC content of the three samples from animals in the experimental group was remarkably different from that of the three animals in the control group, indicating the quality of RNA sequencing. A quality score of 20 and 30 (Q20 and Q30, respectively) indicates that the detection accuracy of the inferred basis was 97.81% and 94.19%, respectively. The results of detailed sequencing are shown in Table 2.

**Table 2:** Results from the RNA-sequencing clean data analysis

Source of sample	Sample	Raw reads	Clean reads	Clean bases	Error (%)	Q20 (%)^a^	Q30 (%)^a^	GC content (%)
Infected animal	M_1	23,038,190	21,613,812	6.48G	0.03	97.79	94.17	52.91
	M_2	23,687,149	22,424,278	6.73G	0.03	97.86	94.2	52.16
	M_3	22,740,572	21,415,289	6.42G	0.03	97.82	94.21	52.87
Control animal	cM_1	23,501,179	22,220,310	6.67G	0.03	97.73	93.98	52.6
	cM_2	21,444,553	20,346,356	6.10G	0.03	97.73	94.04	53.5
	cM_3	23,479,997	21,996,776	6.60G	0.02	97.93	94.53	52.93

^a^Q20, quality score of 20, representing an error rate of 1 in 100; Q30 indicates that all of the reads will be perfect, with no errors or ambiguities

**Fig. 1:**
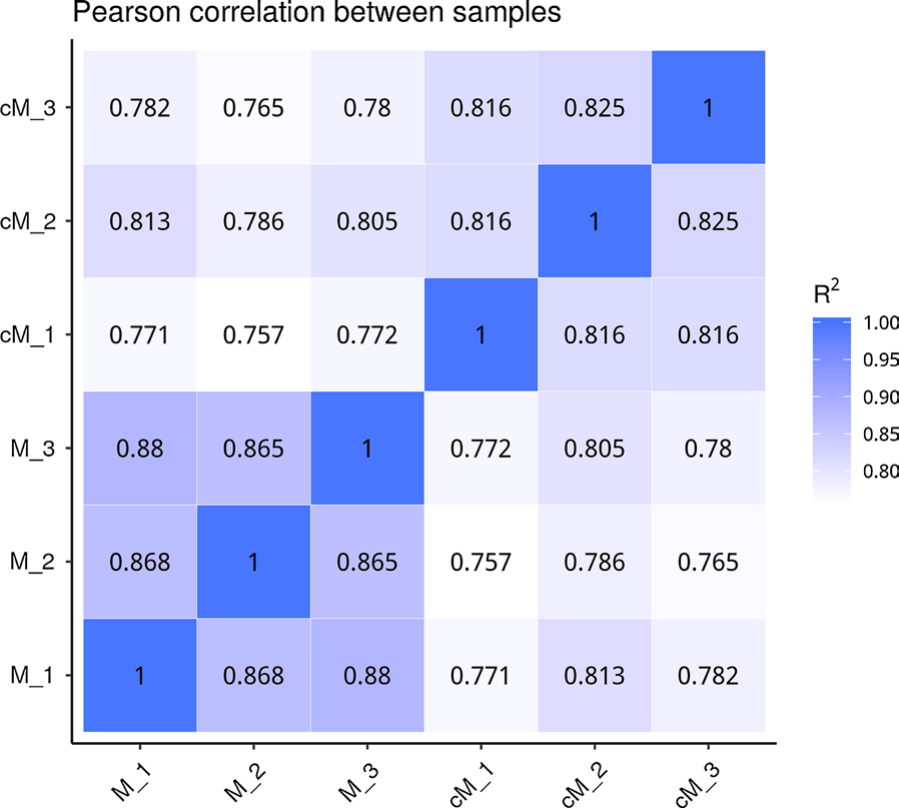
Heat map showing the size of the Pearson correlation coefficient (*R*^2^) matrix between different groups. The abscissa is the log10 (FPKM + 1) of sample 1, and the ordinate is the log10 (FPKM + 1) of sample 2. FPKM, Fragments per kilobase of exon per million mapped fragments

The Pearson correlation coefficient of gene expression at different time points was close to 1, indicating a high similarity of gene expression patterns among samples (Fig. 1).

**Fig. 2:**
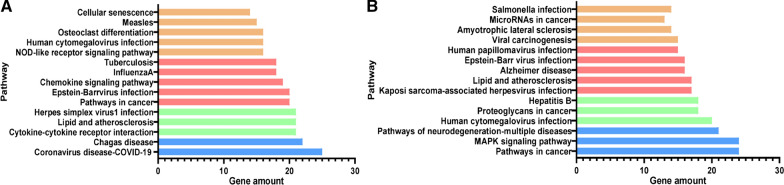
KEGG pathway analysis bar graph of the top 15 differentially expressed genes (DEGs) in terms of enrichment degree. The* X*-axis shows gene amount, and the* Y*-axis corresponds to the KEGG pathway. **A** Upregulated gene enrichment pathway, **B** downregulated gene enrichment pathway. KEGG Kyoto Encyclopedia of Genes and Genomes

A total of 2808 up- or downregulated DEGs of *T. gondii*-infected masked civets were transformed into differential genes of BALB/c mice, and a total of 651 differential genes were obtained (Additional file 1: Table S1) and compared with data from BALB/c mice *T. gondii*-infected (PRJNA702090) (Additional file 2: Table S2). Through the comparison, a total of 304 DEGs with the same expression patterns (Additional file 3: Table S3) were identified. The KEGG pathway analysis shows the most commonly upregulated (Fig. 2a) and downregulated DEGs (Fig. 2b) in the enrichment pathways. These results indicated that some of the differential genes of *T. gondii-*infected masked civet also had the same expression pattern in *T. gondii* BALB/c mice.

**Fig. 3:**
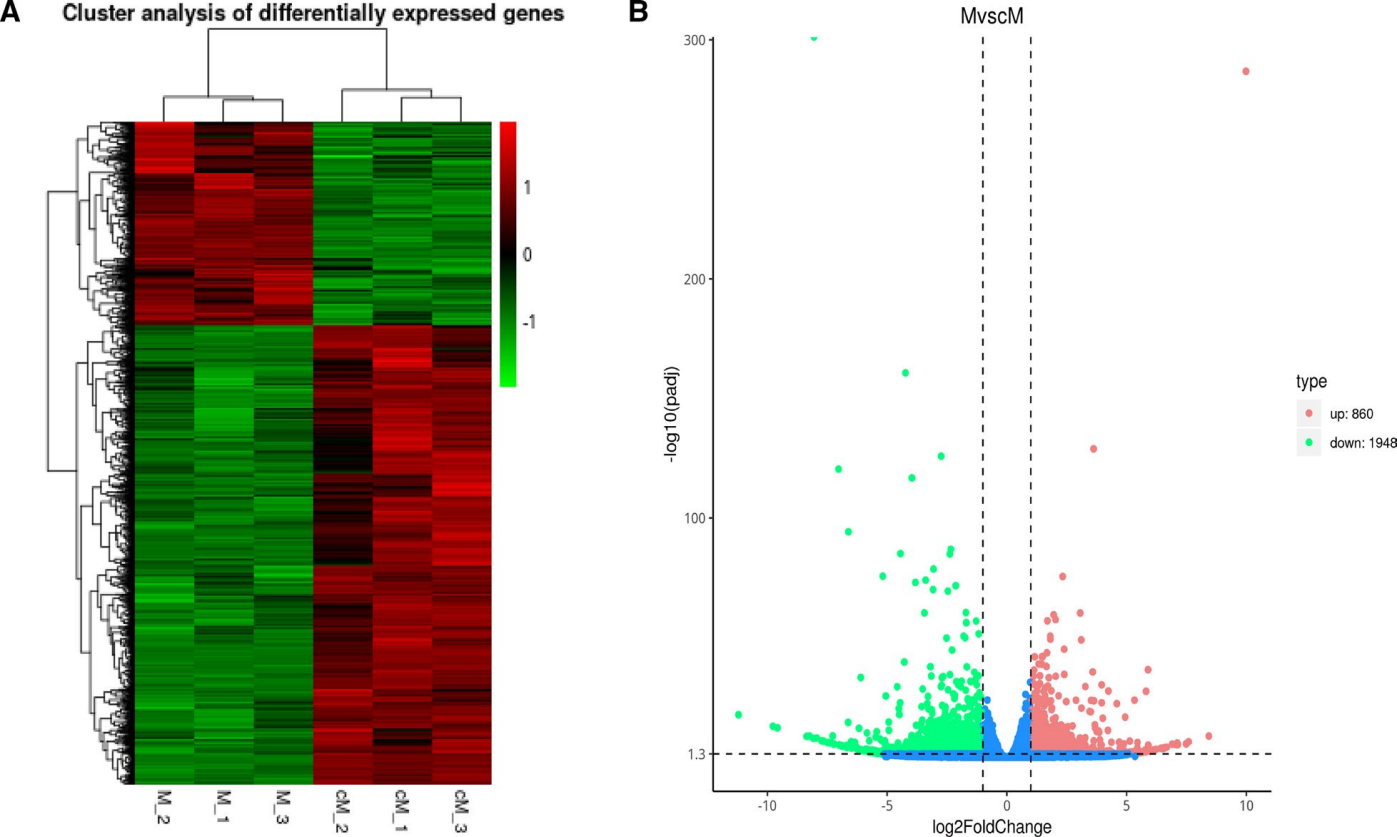
RNA sequencing and transcript differential expression analysis. **A** Clustering gene heat map. The ordinate is the clustering result of DEGs; the abscissa represents the trial number. **B** Volcano map of DEGs. Red represents upregulated DEGs, and green represents downregulated DEGs

**Fig. 4:**
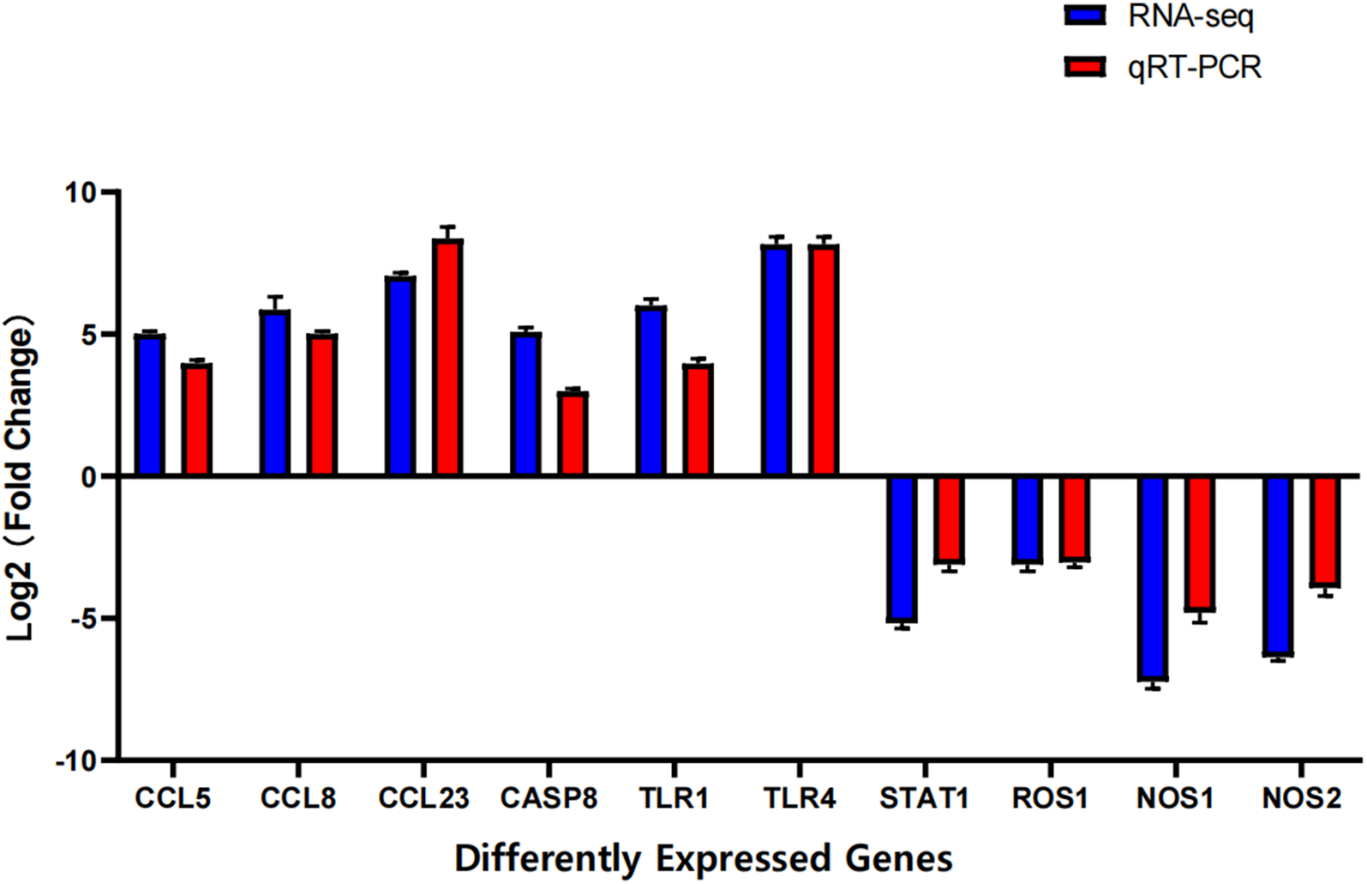
qRT-PCR verifies the results of RNA-sequencing. The* X*-axis represents the gene’s name, and the* Y*-axis represents the relative expression of the gene. DEGs above the horizontal line are upregulated and those below the horizontal line are downregulated. qRT-PCR Quantitative reverse-transciption PCR

Transcriptome data are represented in Fig. 3a as a cluster map and in Fig. 3b as a volcano map. The results showed an obvious difference between the infected animals and the control animals, and the difference between biological repeats DEGs was small (*P*-value < 0.05; log2 FC > 1.5). Ten differentially expressed genes and their expression levels were verified by qRT-PCR, and the results of qRT-PCR were consistent with those of RNA-sequencing (RNA-Seq), which proved the authenticity and genuine validity of the sequencing data (Fig. 4).

### GO and KEGG enrichment analysis

GO functional enrichment and KEGG pathway analysis were performed on transcription data on DEGs from samples from infected animals and control animals.

**Fig. 5:**
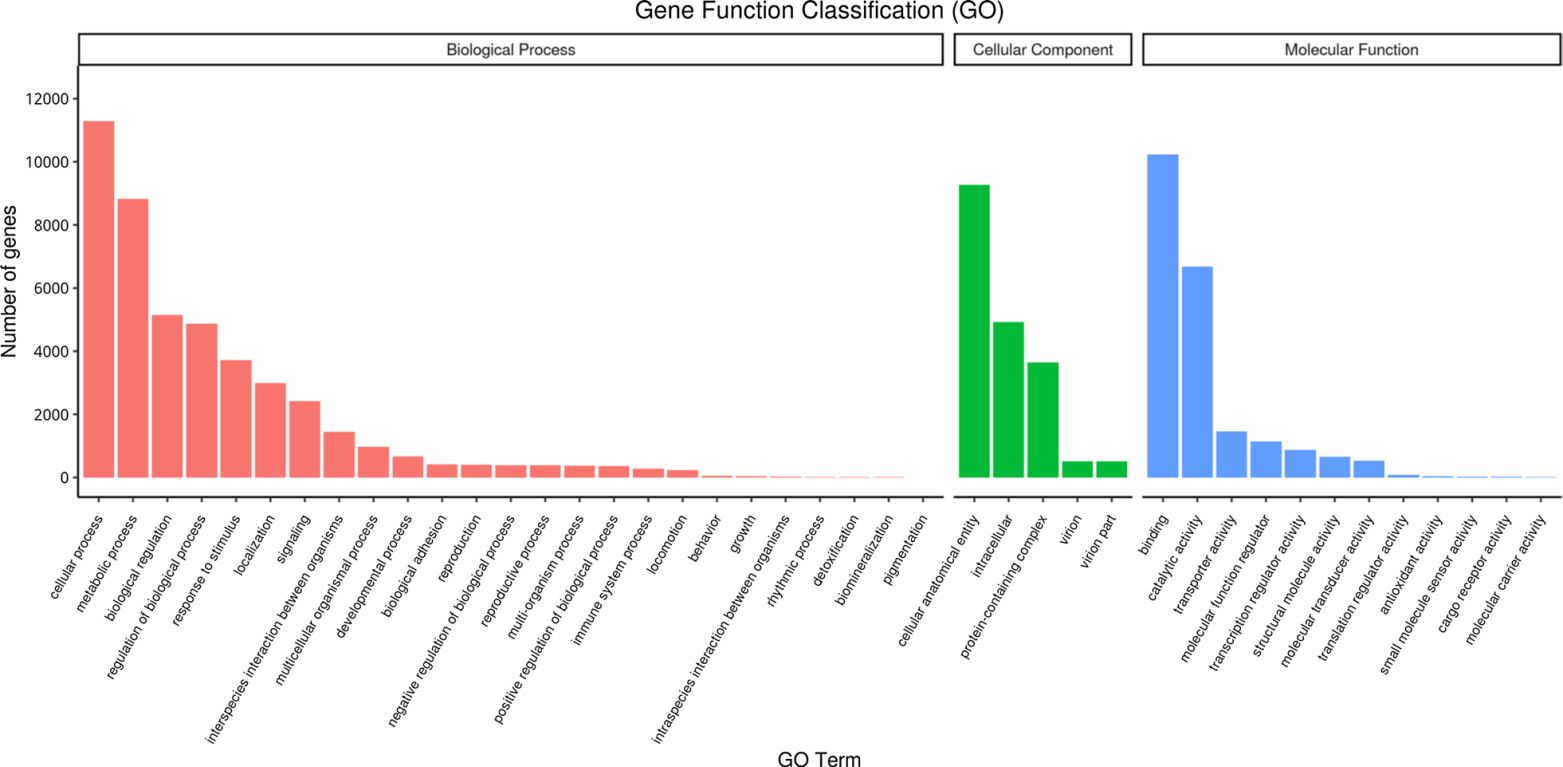
The GO analysis of the DETs. The GO is distributed into three main categories: biological process, cellular component and molecular function. The* X*-axis represents the GO term corresponding to the gene, and the* Y*-axis represents the number of genes. GO Gene ontology

The GO functional annotations analysis explores the biological functions of DEGs. GO annotations include bBiological processes (BP), cellular components (CC) and molecular function (MF). All DEGs were detected in the brain tissues of chronically infected masked civet. A statistical column chart was drawn to reflect the analysis results of the enriched GO term (Fig. 5). The cell treatment process, metabolic process, cell differentiation and biological regulation were the major vital processes in terms of biological functions.

**Fig. 6:**
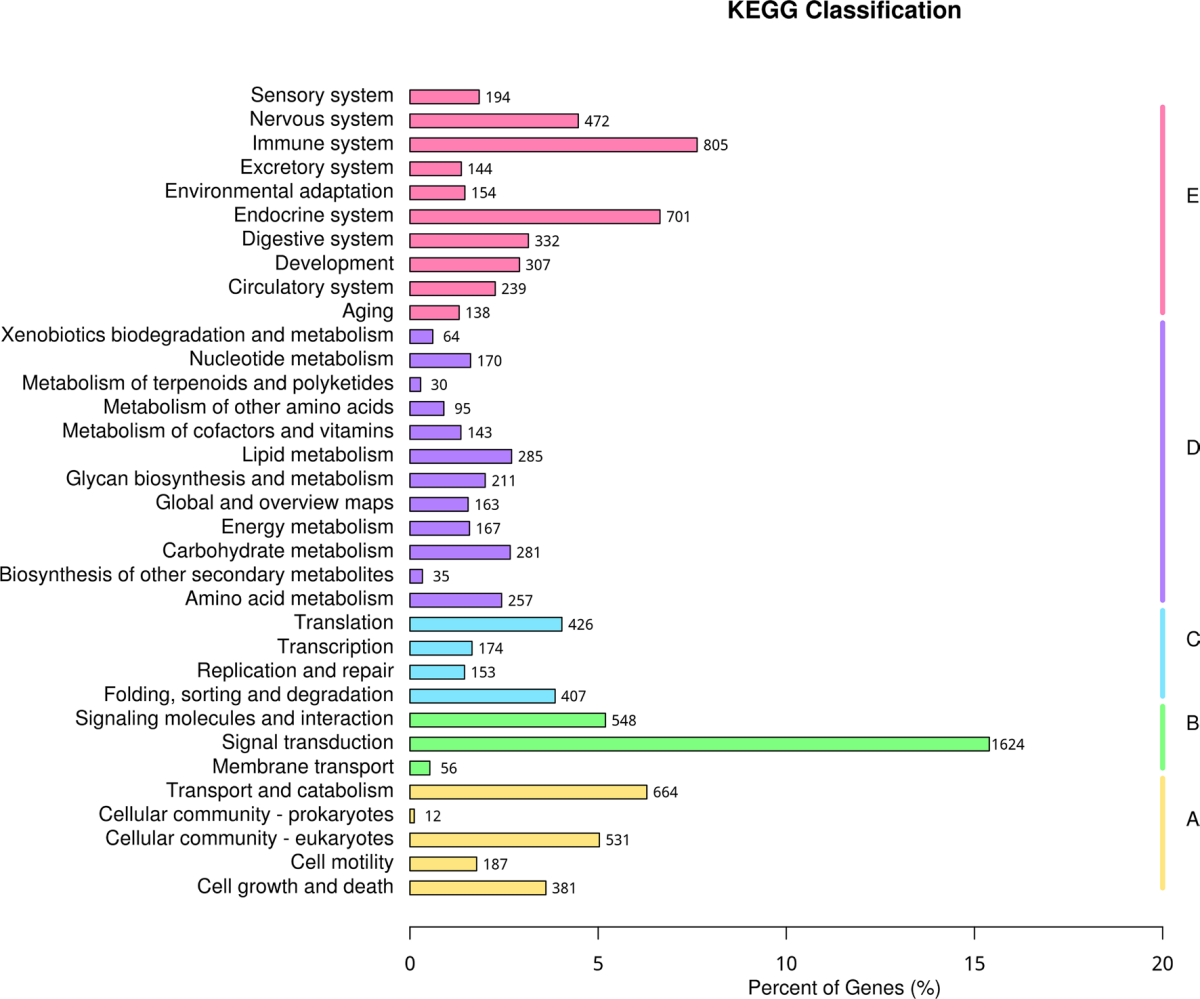
Pathway analysis of DEGs in KEGG. Differential gene function enrichment pathways mainly involve signal transduction, the immune system, the endocrine system and transport and catabolism

**Fig. 7:**
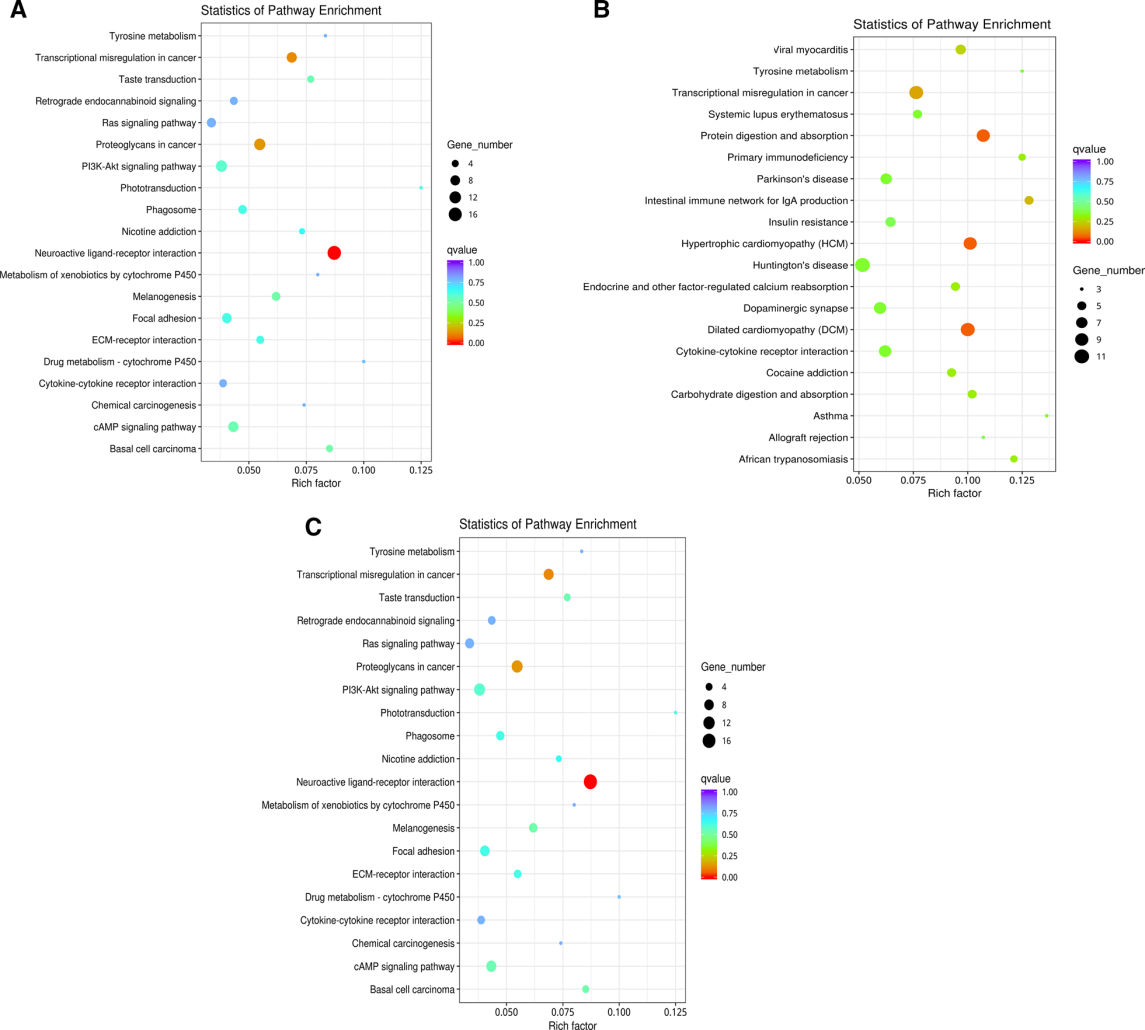
Scatter map of the top 20 enriched pathways in the KEGG pathway analysis. The* X*-axis shows enrichment factors, and the* Y*-axis corresponds to the KEGG pathway. **A** Upregulated gene enrichment pathways, **B** downregulated gene enrichment pathways, **C** pathway enrichment analysis of overall genes

The results of the KEGG functional annotation analysis of DEGs is shown in Fig. 6. The KEGG database was utilized to analyze pathway enrichment. DEGs of each group were further explored in those pathways that play an essential role in chronic infection of *T. gondii* and then plotted to scatter maps. In total, 168 pathways were found to be enriched with upregulated DEGs. The pathway maps of the top 20 gene enrichment levels was screened. (Fig. 7a). These include neuroactive ligand-receptor interaction, proteoglycans in cancer, transcriptional downregulation in cancer and melanogenesis. Among the downregulated DEGs, 194 pathways were enriched, and the top 20 pathways with the highest enrichment were screened out (Fig. 7b). These include dilated cardiomyopathy (DCM), hypertrophic cardiomyopathy (HCM), protein digestion and absorption and transcriptional downregulation in cancer. Lastly, all KEGG pathways of all DEGs were analyzed. The top 20 pathways were screened out (Fig. 7c), including, transcriptional downregulation in cancer, neuroactive ligand-receptor interaction, protein digestion and absorption, viral myocarditis, among others.

The GO and KEGG analysis revealed that DEGs were abundant in the immune pathway. The chemokine signaling pathway, Toll-like receptor (TLR) signaling pathway, JAK-STAT signaling pathway, T cell receptor signaling pathway and PI3K-Akt signaling pathways were all enriched. In terms of the immune system, 10 essential driver genes were screened:* CCL5*,* CCL8*,* CCL23*,* CASP8*,* TLR1*,* TLR4*,* STAT1*,* ROS1*,* NOS1* and* NOS2* (Table 3).

**Table 3:** Partial list of differentially expressed genes in *Toxoplasma gondii*-infected masked civet brain

Gene symbol	Full name of gene	Log_2_ (FC)	Differential expression
*CCL8*	C–C motif chemokine ligand 8	6.5608	Upregulated
*CCL23*	C–C motif chemokine ligand 23	6.17868	Upregulated
*TLR1*	Toll-like receptor 1	5.61362	Upregulated
*TLR4*	Toll-like receptor 4	1.53097	Upregulated
*CCL5*	C–C motif chemokine ligand 5	1.29567	Upregulated
*CASP8*	Caspase 8	1.11099	Upregulated
*NOS2*	Nitric oxide synthase 2	− 5.60721	Downregulated
*STAT1*	Signal transducer and activator of transcription 1	− 3.40022	Downregulated
*ROS1*	ROS proto-oncogene 1	− 3.41519	Downregulated
*NOS1*	Nitric oxide synthase 1	− 1.60721	Downregulated

*FC* Fold change

## Discussion

According to previous reports, *T. gondii* changes the behavior of its intermediate host due to the invasion of parasite cysts into the brain tissue. In one study, infected rodents were found to have decreased learning and memory, but increased activity [30]. In humans, *T. gondii* infection can also cause neurodegenerative diseases, such as mental disorders, schizophrenia and depression [31].

In this study, RNA-Seq analysis was performed on brain tissues extracted from masked civets chronically infected with *T. gondii*. Comparison of the results from the infected and control groups revealed 2808 DEGs, of which 860 were upregulated and 1948 were downregulated. We analyzed the pathway enrichment of all transcripts based on the GO and KEGG databases with the aim to evaluate the biological correlation of selected genes. Screening of the DEGs was related to the immune system. The chemokine signaling pathway, the TLR signaling pathway, T cell receptor signaling pathway, NOD-like receptor signaling pathway and JAK-STAT signaling pathway are actual examples of immune pathways with high gene enrichment.

*Toxoplasma gondii* infection is thought to damage the brain tissue and is a risk factor for neurological and mental disorders. Recently, the chemokine system involved in the immune response has been considered a vital element of signal transduction and neural function in brain cells [32]. Previous analyses of the KEGG pathway revealed that chronic infection of *T. gondii* can cause enhanced chemokine signaling [33, 34], and occasionally release chemokines through inflammatory cytokines such as interleukin-1 (IL-1) stimulation [35], Chemokines play a role by binding to different types of chemokine receptors on the surface of cell membranes, thereby helping to inhibit *T. gondii* infection. CCL5, CCL8 and CCL23 belong to the CC chemokine subfamily, and CCL5, CCL10, and CCL23 are also critical chemokines that attract mononuclear macrophages into inflammatory sites [36, 37].

Interferon gamma (IFN-γ) is an important cytokine that protects host cells from *T. gondii* invasion. Indoleamine-2 indoleamine 2,3-dioxygenase can be induced to convert tryptophan into l-formyluridine, which is necessary for the development and proliferation of *T. gondii*. Thus, the growth and expansion of *T. gondii* are inhibited by tryptophan starvation during the primary metabolism [38, 39]. Some studies have shown that STAT1 transcription factors mediate the IFN-γ response, which is essential for host defense against intracellular pathogens such as *T. gondii*. However, a previous infection with *T. gondii* can inhibit this response [40]. Previous studies have shown that mice without the IFN-γ receptor or the signal transducer and activator of the STAT1 gene are highly susceptible to *T. gondii* after inoculation with non-lethal *T. gondii* strains [41]. Furthermore, the inhibitor of STAT1 transcription (IST) plays a key role in limiting IFN-γ signaling in bradyzoites. The export of bradyzoite protein protects host cells from IFN-γ-mediated cell death, even when the export is restricted to latent stages [42]. In the present study, the genes encoding STAT1, NOS1, NOS2 and ROS1, all related to immunity, were simultaneously screened. Inhibition of STAT1 transcriptional activity can downregulate the expression of major histocompatibility complex and inducible nitric oxide synthase (iNOS), which is helpful for the survival of *T. gondii*. IFN-γ-induced iNOS and activated oxygen (reactive oxygen species) effector molecules can effectively resist the *T. gondii* invasion [43, 44]. PI3K-Akt signal transduction plays a vital role in *T. gondii* invasion of host cells because phosphatidylinositol (PIP3) accumulates rapidly in host cells in response to the infective tachyzoites. More importantly, PI3K inhibitors partially reduce parasite entry [45]. Akt can regulate various cell processes; for example, it can increase metabolism, control growth and synthesis and inhibit apoptosis. Therefore, we can infer that the PI3K-Akt signal pathway has an inhibitory effect on *T. gondii* invasion of masked civet and other wild species.

**Fig. 8:**
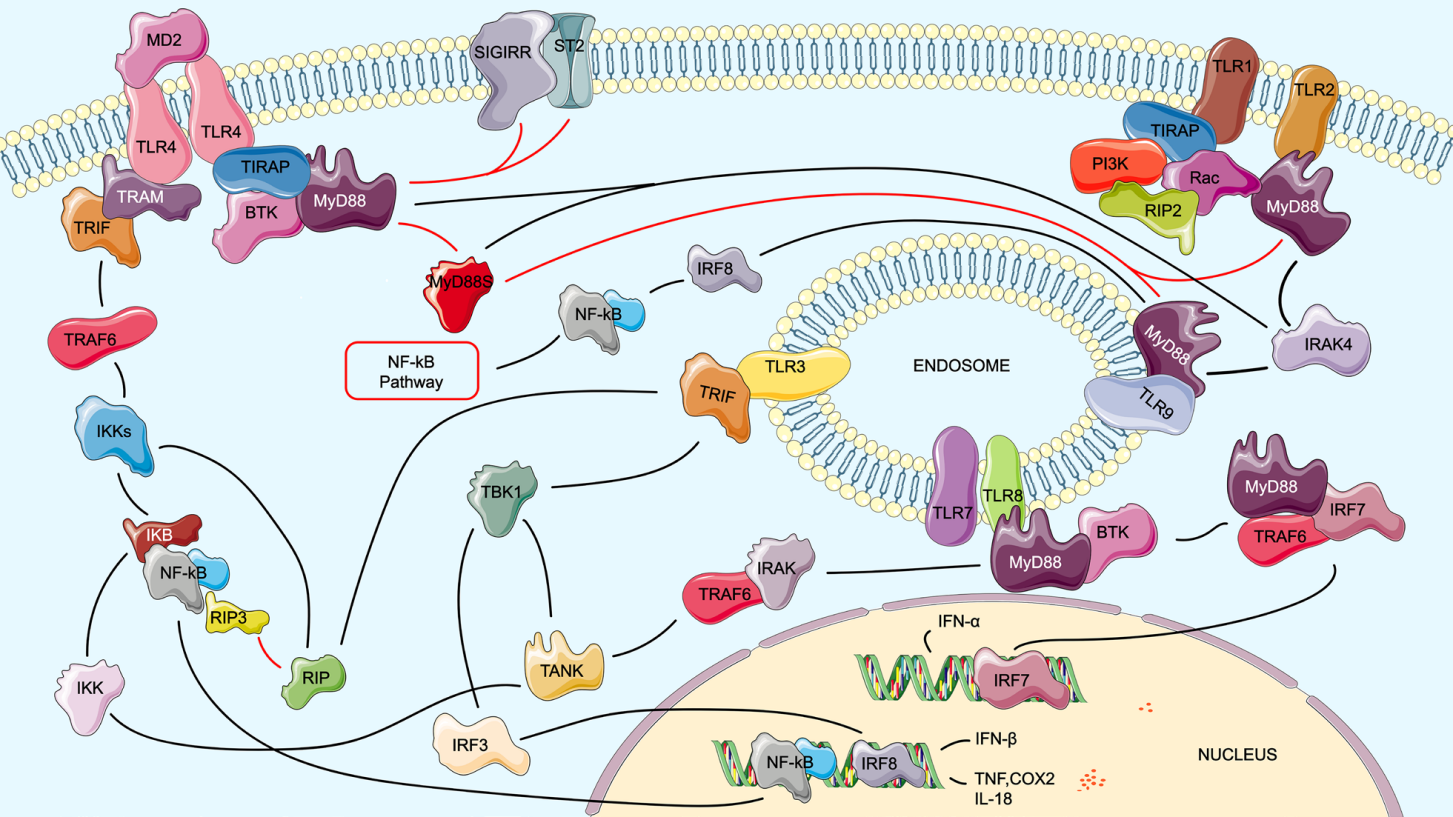
TLR1- and TLR4-mediated activation of the body generates cellular immune responses through activation of the NF-κB signaling pathway, which can defend against the *T. gondii* invasion of the host. TRL, Toll-like receptor

TLR signaling pathways can participate in non-specific immunity. Activation of the signal transduction pathway (Fig. 8), triggering the body to generate immune cell response and inducing the expression of inflammatory factors are important and effective mechanisms against *T. gondii* invasion of host cells [46, 47]. Previous studies have shown that the TLR signaling pathway plays a vital role in host resistance to *T. gondii* and its pathogenesis infection (Fig. 8). TLR1 and TLR4 may be expressed in T cells, mononuclear macrophages and lymphocytes and NK cells during *T. gondii* invasion. *Toxoplasma gondii* can also activate TLR4 to induce inflammation and mediate neuronal apoptosis, which plays a vital role in the inflammatory response induced by brain injury [48, 49]. We also screened the CASP8 gene for inflammatory body-mediated cell death, which is an essential pathway in controlling *T. gondii* host cell death [50]. It has been reported that CASP8 plays an indispensable role in regulating inflammation and can also act as an antigen to activate NF-kB and promote cytokine production [51].

## Conclusion

In the present study, brain tissue samples were collected from masked palm civet chronically infected with *T. gondii* for transcriptome analysis, functional annotation and collateral analysis of differential genes. The immune pathways of highly enriched differential genes were screened; these mainly involved the chemokine signaling pathway, TLR signaling pathway, JAK-STAT signaling pathway, T cell receptor signaling pathway and PI3K-Akt signaling pathway. The immune pathway reported in this study may provide insights into the mechanism of the immune response to *T. gondii* chronic infection in wild species. The screened genes can be used as target genes to develop drugs to treat *T. gondii-*infected wild animals and, subsequently, implement effective prophylactic measures.

## Supplementary Information

**Additional file 1: Table S1.** Masked civet differential genes transformation in BALB/c mice differential gene.

**Additional file 2: Table S2.*** T. gondii*-infected BALB/c mice differential genes

**Additional file 3: Table S3.** Masked civet and BALB/c mice co-expressed differential genes

## Data Availability

The sequencing data generated in this study have been deposited into BioProject (accession no. PRJNA760987, https://www.ncbi.nlm.nih.gov/bioprjet/PRJNA760987).

## Abbreviations

CASP8: Caspase-8
CCL: C-C motif chemokine ligand
DEGs: Differentially expressed genes
GO: Gene ontology
KEGG: Kyoto Encyclopedia of Genes and Genomes
IFN-γ: Interferon gamma
iNOS: Inducible nitric oxide synthase
qRT-PCR: Quantitative reverse-transcription PCR
ROS: Reactive oxygen species
STAT: Signal transducer and activator of transcription
TLR: Toll-like receptor
